# Systemic arteriosclerosis and eating behavior in Japanese type 2 diabetic patients with visceral fat accumulation

**DOI:** 10.1186/s12933-015-0174-7

**Published:** 2015-01-16

**Authors:** Shiro Fukuda, Ayumu Hirata, Hitoshi Nishizawa, Hirofumi Nagao, Susumu Kashine, Takekazu Kimura, Kana Inoue, Yuya Fujishima, Masaya Yamaoka, Junji Kozawa, Tetsuhiro Kitamura, Tetsuyuki Yasuda, Norikazu Maeda, Akihisa Imagawa, Tohru Funahashi, Iichiro Shimomura

**Affiliations:** Department of Metabolic Medicine, Graduate School of Medicine, Osaka University, 2-2 B-5, Yamada-oka, Suita, Osaka 565-0871 Japan; Department of Metabolism and Atherosclerosis, Graduate School of Medicine, Osaka University, 2-2-B, Yamada-oka, Suita, Osaka 565-0871 Japan

**Keywords:** Type 2 diabetes, Visceral fat accumulation, Adiponectin, Systemic arteriosclerosis, Vascular ultrasonography, Eating behavior

## Abstract

**Background:**

Visceral fat accumulation is a major etiological factor in the progression of type 2 diabetes mellitus and atherosclerosis. We described previously visceral fat accumulation and multiple cardiovascular risk factors in a considerable number of Japanese non-obese subjects (BMI <25 kg/m^2^). Here, we investigated differences in systemic arteriosclerosis, serum adiponectin concentration, and eating behavior in type 2 diabetic patients with and without visceral fat accumulation.

**Methods:**

The study subjects were 75 Japanese type 2 diabetes mellitus (age: 64.8 ± 11.5 years, mean ± SD). Visceral fat accumulation represented an estimated visceral fat area of 100 cm^2^ using the bioelectrical impedance analysis method. Subjects were divided into two groups; with (n = 53) and without (n = 22) visceral fat accumulation. Systemic arteriosclerosis was scored for four arteries by ultrasonography. Eating behavior was assessed based on The Guideline for Obesity questionnaire issued by the Japan Society for the Study of Obesity.

**Results:**

The visceral fat accumulation (+) group showed significantly higher systemic vascular scores and significantly lower serum adiponectin levels than the visceral fat accumulation (−) group. With respect to the eating behavior questionnaire items, (+) patients showed higher values for the total score and many of the major sub-scores than (−) patients.

**Conclusions:**

Type 2 diabetic patients with visceral fat accumulation showed 1) progression of systemic arteriosclerosis, 2) low serum adiponectin levels, and 3) differences in eating behavior, compared to those without visceral fat accumulation. Taken together, the findings highlight the importance of evaluating visceral fat area in type 2 diabetic patients. Furthermore, those with visceral fat accumulation might need to undergo more intensive screening for systemic arteriosclerosis and consider modifying their eating behaviors.

## Background

Type 2 diabetes mellitus accelerates the process of arteriosclerosis and may result in severe cardiovascular events. Since a variety of etiological factors seem to contribute to type 2 diabetes mellitus, it is important to understand the etiology in each patient and detect potential arteriosclerosis at an early stage. In type 2 diabetes, arteriosclerosis is a polyvascular and multifocal disease that can advance without overt symptoms, making early prediction of coronary artery diseases (CAD) important for the prevention of cardiovascular events in these patients. We have recently reported that quantification of the severity of arteriosclerosis by vascular ultrasonography is a useful tool for predicting CAD, and that the metabolic syndrome was a significant determinant of total systemic vascular score of ≥2 [1].

Recently, the prevalence of obesity-related type 2 diabetes mellitus has increased worldwide, especially in Asia [2-5]. We reported previously that a considerable proportion of non-obese (body mass index (BMI) <25 kg/m^2^) Japanese subjects have visceral fat accumulation (visceral fat area (VFA) ≥100 cm^2^), as well as multiple risk factors of cardiovascular diseases, and that reduction of VFA was significantly associated with a decrease in total cardiovascular risk [6]. Patients with visceral fat accumulation also show dysregulation of adipocytokines, such as hypoadiponectinemia, which is associated with type 2 diabetes mellitus and CAD [7-9]. Adiponectin is an adipose-specific endocrine factor that exhibits anti-diabetic, anti-atherogenic, and anti-inflammatory properties. It is therefore possible that type 2 diabetic patients with visceral fat accumulation may be more affected by arteriosclerosis, although there is little evidence to confirm or deny this hypothesis.

In modifying the lifestyle of patients with obesity, cognitive behavioral therapy is important in addition to diet and exercise therapy [10], and research findings show a relationship between eating behavior and obesity/weight gain [11]. It is therefore important to help each patient identify and improve eating behavior problems. In the present study, we investigated differences in the clinical features of Japanese type 2 diabetic patients with and without visceral fat accumulation, focusing on systemic arteriosclerosis, serum adiponectin concentration, and eating behavior.

## Methods

### Subjects

The study subjects were selected from April 2012 to December 2012 among inpatients hospitalized for the control type 2 diabetes at the Division of Endocrinology and Metabolism of Osaka University Hospital, and outpatients who visited the “Diabetes & Metabolic Station” outpatient clinic of Osaka University Hospital. Written consent was obtained from each subject after explaining the purpose and possible complications of the study. This study complied with the Guidelines of the Ethnical Committees of Osaka University. Type 2 diabetes was defined according to the World Health Organization (WHO) national diabetic group criteria of 2006 and/or treatment of diabetes. The following patients were excluded: 1) patients in whom waist circumference (WC) and estimated visceral fat area (eVFA; see ‘Clinical examination’) was not measured, 2) patients who were not clinically diagnosed with type 2 diabetes mellitus.

### Clinical examination

Height, weight, and WC were measured in the standing position (in inpatients, these data were measured on admission). The bioelectrical impedance analysis method was used to measure eVFA, which we showed previously to correlate significantly with VFA determined by computed tomography [12]. The duration of diabetes was retrieved through medical interview. Systolic/diastolic blood pressure (BP) was measured with a standard mercury sphygmomanometer on the right arm in the supine position after at least 10 minutes of rest. Venous blood samples were collected in the morning after overnight fasting for measurements of glucose and HbA1c (National Glycohemoglobin Standardization Program (NGSP)), serum C-peptide, lipids, creatinine, and uric acid (UA). Estimated glomerular filtration rate (eGFR) was calculated using the simplified Modification of Diet in Renal Disease equation modified by the appropriate coefficient for Japanese populations by gender [13]. The serum samples obtained at baseline from each participant were stored promptly at −20°C. After thawing, serum levels of total adiponectin in 62 patients were measured by enzyme-linked immunosorbent assay (ELISA) (human adiponectin ELISA kit, Otsuka Pharmaceutical Co. Tokushima, Japan), as reported previously [14]. Urinary albumin-creatinine ratio (uACR) was calculated from a single spot urine specimen collected in the morning. The maximum or mean intima-media thickness (IMT) of the common carotid artery was measured in supine position by echography as described previously [15,16].

### Definition of visceral fat accumulation, diabetic retinopathy, diabetic nephropathy, hypertension, dyslipidemia, and metabolic syndrome

Visceral fat accumulation was defined as eVFA ≥100 cm^2^ [17,18]. Diabetic retinopathy was assessed by an ophthalmologist, and based on this we divided patients into two groups (NDR and SDR/PrePDR/PDR). Diabetic nephropathy was diagnosed when the uACR was ≥30 mg/g creatinine. Hypertension was defined by systolic BP ≥140 mmHg and/or diastolic BP ≥90 mmHg. Dyslipidemia was defined as low-density lipoprotein cholesterol (LDL-C) concentrations ≥140 mg/dl, triglyceride (TG) concentrations ≥150 mg/dl, and/or high-density lipoprotein cholesterol (HDL-C) concentrations ≥40 mg/dL. Patients were considered positive for hypertension and/or dyslipidemia if they received antihypertensive and/or anti-dyslipidemic medications, respectively. Metabolic syndrome was defined as follows: 1) eVFA ≥100 cm^2^ and 2) positive for hypertension or dyslipidemia (except for the criteria of LDL-C). This definition was based on the Japanese guidelines for metabolic syndrome [19], using eVFA instead of WC.

### Questionnaire for eating behavior

Eating behavior was assessed in patients by using the questionnaire of The Guideline For Obesity issued by the Japan Society for the Study of Obesity, as in our previous reports [20,21]. Briefly, this questionnaire comprises 55-item questions of seven major scales as follows: 1) recognition for weight and constitution, 2) external eating behavior, 3) emotional eating behavior, 4) sense of hunger, 5) eating style, 6) food preference, and 7) regularity of eating habits. All items were rated on a four-point scale ranging from 1 (seldom) to 4 (very often).

### Evaluation of systemic vascular score

Among all study patients, 67 subjects were scored as having systemic arteriosclerosis by vascular ultrasonography, as per our previous report [15]. Briefly, we qualitatively evaluated the presence of systemic arteriosclerosis by systemic vascular ultrasonography using an ultrasound scanner, evaluating the vessel interior of the following four arteries: 1) common carotid arteries (for existence of plaque, calcification), 2) renal arteries (stenosis), 3) abdominal aorta between the diaphragm and bifurcation of the common iliac arteries (plaque, calcification, aneurysm), and 4) common iliac arteries (plaque, calcification). Plaques in the aortic and common iliac artery walls were considered present when the intimal surface was not smooth and >1 mm in thickness. Renal arterial stenosis was diagnosed by a peak systolic velocity of more than 200 cm/sec with post-stenotic turbulence or renal aortic flow velocity ratio. The score was set as 1 for an abnormality on either side in carotid, renal, and common iliac arteries (0 to 4).

### Statistical analysis

Data are presented as mean ± SD. First, Shapillo-Wilk’s W test was used to determine the distribution of each parameter. Next, the difference between groups in systolic BP, diastolic BP, LDL-C, HDL-C, and UA (i.e., variables with normal distribution) were analyzed with Welch’s *t*-test. For the other parameters (variables with skewed distribution), Mann-Whitney’s *U* test was used. We performed the Cochran-Armitage trend test to analyze relationships between the systemic vascular score groups. Fischer’s exact test was used to compare gender, frequencies of diabetic retinopathy, nephropathy, hypertension, and dyslipidemia, and percentage of patients with systemic vascular score exceeding 2. In all cases, probability (*P*) values of <0.05 were considered statistically significant. All analyses were performed with the JMP Pro 10.0.2 for Windows (SAS Institute, Cary, NC).

## Results

### Participant characteristics

We screened 92 Japanese patients with type 2 diabetes (75 inpatients and 17 outpatients), excluded 17 patients (they did not have eVFA data), and finally enrolled 75 patients in this study (58 inpatients and 17 outpatients; 41 males and 34 females; age, 64.8 ± 11.5 years; BMI 26.4 ± 5.8 kg/m^2^, WC; males 95 ± 14.4 cm, females 93.3 ± 11.9 cm; eVFA; males 151.4 ± 73.0 cm^2^, females 118.9 ± 55.5 cm^2^). The enrolled patients were divided into two groups based on eVFA. We defined the 53 patients (32 males and 21 females, 42 inpatients and 11 outpatients) with an eVFA ≥100 cm^2^ as the visceral fat accumulation (+) group, and the 22 remaining patients (9 males and 13 females, 16 inpatients and 6 outpatients) as the visceral fat accumulation (−) group. Table 1 summarizes the baseline characteristics of the patients.

**Table 1:** **Baseline characteristics**

**Variable**	**All**	**Visceral fat accumulation**		***P*** **value**
		**(+) group**	**(−) group**	
n (Males/Females)	75 (41/34)	53 (32/21)	22 (9/13)	0.14^**^
Age (years)	64.8 ± 11.5	63.0 ± 12.7	69.1 ± 6.2	0.09^+^
Body weight (kg)	69.2 ± 19.7	75.8 ± 19.5	53.1 ± 7.2	**<0.001** ^+^
Body mass index (kg/m^2^)	26.4 ± 5.8	28.5 ± 5.4	21.3 ± 2.3	**<0.001** ^+^
Waist circumferences (cm), all	94.2 ± 13.2	100.0 ± 11.5	81.3 ± 6.7	**<0.001** ^+^
Males	95 ± 14.4	99.4 ± 12.9	79.3 ± 5.1	**<0.001** ^+^
Females	93.3 ± 11.9	100.0 ± 9.1	82.7 ± 7.5	**<0.001** ^+^
eVFA (cm^2^), all	136.7 ± 7.8	164.4 ± 60.0	70.0 ± 21.0	**<0.001** ^+^
Males	151.4 ± 73.0	174.2 ± 66.0	70.7 ± 18.7	**< 0.001** ^+^
Females	118.9 ± 55.5	149.4 ± 46.9	69.5 ± 23.3	**< 0.001** ^+^
Duration of diabetes (years)	14.6 ± 10.5	12.7 ± 9.1	19.1 ± 12.4	**0.04** ^+^
Diabetic retinopathy	n = 19 (25%)	11 (21%)	8 (36%)	0.24^*^
Diabetic nephropathy	n = 23 (31%)	18 (34%)	3 (14%)	0.27^*^
Hypertension	n = 56 (75%)	39 (74%)	17 (77%)	1.00^*^
Dyslipidemia	n = 58 (77%)	45 (85%)	13 (59%)	**0.03** ^*^
Metabolic syndrome	n = 52 (69%)	52 (98%)	-	
Systolic BP (mmHg)	128.1 ± 15.5	129.6 ± 15.6	124.5 ± 14.8	0.20^#^
Diastolic BP (mmHg)	73.9 ± 12.0	75.3 ± 12.0	70.7 ± 11.4	0.12^#^
Glucose (mg/dl)	150.1 ± 47.0	153.0 ± 49.0	142.9 ± 42.0	0.44^+^
HbA1c (NGSP) (%)	8.7 ± 1.7	8.8 ± 1.7	8.5 ± 1.9	0.33^+^
serum C-peptide (ng/ml)	2.2 ± 1.7	2.5 ± 1.8	1.4 ± 0.7	**0.005** ^+^
T-Cho (mg/dl)	190.2 ± 40.5	194.0 ± 44.4	181.1 ± 27.8	0.35^+^
TG (mg/dl)	170.7 ± 233.8	199.3 ± 273.4	103.1 ± 40.1	**0.001** ^+^
LDL-C (mg/dl)	105.6 ± 33.2	108.4 ± 36.0	99.0 ± 24.8	0.20^#^
HDL-C (mg/dl), all	50.2 ± 14.6	46.5 ± 12.4	58.9 ± 15.9	**0.003** ^#^
UA (mg/dl)	5.5 ± 1.5	5.7 ± 1.6	5.0 ± 1.1	**0.03** ^#^
eGFR (ml/min/1.73m^2^)	72.9 ± 19.6	73.9 ± 21.3	70.5 ± 15.1	0.42^+^
uACR (mg/gCr)	67.8 ± 147.8	72.6 ± 167.4	56.1 ± 84.0	0.97^+^
CCA max IMT (mm)	1.80 ± 0.80	1.75 ± 0.79	1.92 ± 0.82	0.39^+^
CCA mean IMT (mm)	0.98 ± 0.30	0.99 ± 0.32	0.98 ± 0.25	0.89^+^
Medications				
for diabetes				
Sulfonylureas	n = 36 (48%)	26 (49%)	10 (45%)	0.81^*^
Glinides	n = 6 (8%)	4 (8%)	2 (9%)	1.00^*^
Biguanides	n = 24 (32%)	18 (34%)	6 (27%)	0.79^*^
Alpha-GIs	n = 14 (19%)	8 (15%)	6 (27%)	0.33^*^
Thiazolidinediones	n = 6 (8%)	4 (8%)	2 (9%)	1.00^*^
DPP-4 inhibitors	n = 22 (29%)	17 (32%)	5 (23%)	0.58^*^
Insulin	n = 15 (20%)	10 (19%)	5 (23%)	0.76^*^
GLP-1 analogs	n = 8 (11%)	7 (13%)	1 (5%)	0.42^*^
for hypertension				
ACEI/ARBs	n = 37 (49%)	26 (49%)	11 (50%)	1.00^*^
for dyslipidemia				
Statins	n = 37 (49%)	26 (49%)	11 (50%)	1.00^*^

Data are mean ± SD [minimum-maximum], or number of subjects (frequency (%)). eVFA; estimated visceral fat area, BP; blood pressure, NGSP; National Glycohemoglobin Standardization Program, T-Cho; total cholesterol, TG; triglyceride, LDL-C; LDL cholesterol, HDL-C; HDL cholesterol, UA; uric acid, eGFR; estimated glomerular filtration rate, uACR; urine albumin-to-creatinine ratio, CCA; common carotid artery, IMT; intima-media thickness, Alpha-GI; alpha-glucosidase inhibitor, DPP-4; dipeptidyl peptidase-4, GLP-1; glucagon-like peptide-1, ACEI; angiotensin-converting enzyme inhibitor, ARB; angiotensin II receptor blocker. **Fisher’s exact test (males vs females), +; Mann-Whitney’s *U* test ((+) group vs (−) group), *Fisher’s exact test ((+) group vs (−) group), #; Welch’s *t*-test ((+) group vs. (−) group).

Compared with the visceral fat accumulation (−) group, the (+) group had significantly shorter duration of diabetes, higher serum C-peptide, higher TG, lower HDL-C, higher UA levels, and higher percentage of dyslipidemia. On the other hand, there were no significant differences in age, sex, BP, glucose, HbA1c, T-Cho, LDL-C, Cr, eGFR, uACR, max IMT, or mean IMT. Furthermore, there were no significant differences between the two groups in the prevalence of diabetic retinopathy, diabetic nephropathy, hypertension, or medications.

### Evaluation of systemic vascular score and serum adiponectin levels

**Figure 1:**
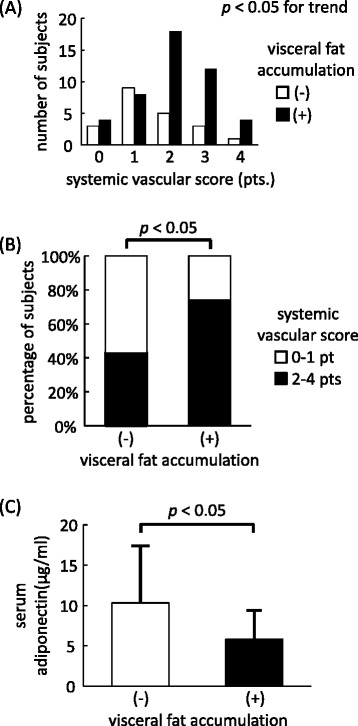
**Visceral fat accumulation and (A) number of subjects, (B) percentage of subjects with systemic vascular score ≥2, (C) serum total adiponectin levels.** Each *P* value is calculated with: **(A)**; Cochran-Armitage trend test, **(B)**; Fischer’s exact test, **(C)**; Mann-Whitney’s *U* test. Data are mean ± SD.

Figure 1 shows the distribution of systemic vascular scores and serum adiponectin levels in each group. We evaluated systemic arteriosclerosis by ultrasonography in 67 subjects (46 of (+) group and 21 of (−) group). The systemic vascular score was significantly higher in the visceral fat accumulation (+) group than in the (−) group (Figure 1A). Furthermore, the percentage of subjects with a systemic vascular score ≥2 was significantly higher in the (+) group than in the (−) group (Figure 1B). Serum adiponectin levels were measured in 62 subjects (44 of (+) group and 18 of (−) group). The visceral fat accumulation (+) group had significantly lower serum adiponectin levels (10.33 ± 7.07 μg/ml) than the (−) group (5.82 ± 3.61 μg/ml) (Figure 1C).

### Assessment of eating behavior

**Figure 2:**
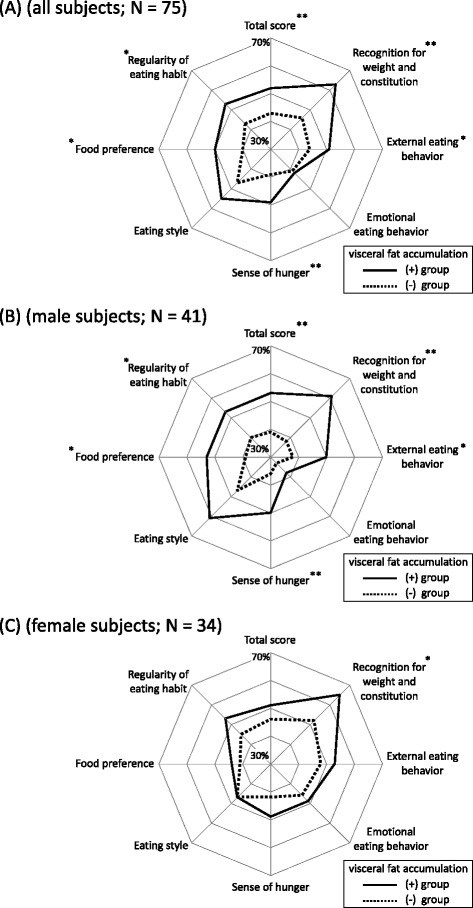
**Comparisons of eating behavior between (+) group (the subjects with visceral fat accumulation, solid line) and (−) group (those without, dotted line), (A) in all subjects, (B) in male subjects, (C) in female subjects.** *; *P* < 0.05, **; *P* < 0.01, (+) group versus (−) group, calculated with Mann-Whitney’s *U* test.

Figure 2 shows a radar chart of eating behavior among all study subjects, with the visceral fat accumulation (+) group (continuous line) showing a significantly higher score than the (−) group (dotted line) in “total score“, “recognition for weight and constitution“, “external eating behavior“, “sense of hunger“, “food preference“, and “regularity of eating habit“ (Figure 2A).

Finally, we examined eating behavior between males (n = 41) and females (n = 34). The differences between the (+) and (−) groups in males were similar to those in all subjects. On the other hand, in females (Figure 2C), only “recognition for weight and constitution” showed a significant difference between (+) and (−) groups.

## Discussion

In this study, type 2 diabetic patients with visceral fat accumulation showed 1) progression of systemic arteriosclerosis, 2) low serum adiponectin levels, and 3) different eating behaviors from those without visceral fat accumulation.

### Systemic arteriosclerosis and serum adiponectin levels in type 2 diabetic patients with visceral fat accumulation

We have shown that systemic arteriosclerosis predicts CAD development in patients with type 2 diabetes [15], and that metabolic syndrome is a determinant of systemic arteriosclerosis [1]. Moreover, hypoadiponectinemia correlates with visceral fat accumulation [22] and predicts the risk of CAD in Japanese type 2 diabetic patients [7]. Adiponectin is an adipocyte-derived plasma protein, which our group identified through a human cDNA project targeting adipose tissue [23] and which shows various anti-atherogenic effects in vascular endothelial cells, smooth muscle cells, and macrophages in cell culture [24-26]. Furthermore, administration of adiponectin with adenovirus vector suppressed the progression of arteriosclerosis in apolipoprotein E-knockout mouse, which is the animal model for arteriosclerosis [27].

Although several research groups reported that adiponectin correlates with diabetes mellitus and/or arteriosclerosis, few studies have compared serum adiponectin levels between type 2 diabetic patients with visceral fat accumulation and those without. The present study therefore provides the first evidence to clarify the state of hypoadiponectinemia in type 2 diabetic patients with visceral fat accumulation, and to suggest that these states together could be associated with the progression of systemic arteriosclerosis. Hypoadiponectinemia is associated with type 2 diabetes, metabolic syndrome, and atherosclerosis [28]. Recently, we reported that C1q-binding adiponectin levels were high in patients with ACS, suggesting the possibility of a protective role of adiponectin against activated-complement system in ACS patients. On the other hand, it is reported that high serum adiponectin is correlated with cardiovascular mortality [29]. The patients with heart failure or renal failure had also high plasma adiponectin levels [30,31]. It is possible that the plasma adiponectin levels were affected by various pathophysiological conditions. Further studies are needed to clarify the association between plasma adiponectin levels and clinical diseases.

In the present study, we found that 45% of the non-obese (BMI <25 kg/m^2^) subjects had visceral fat accumulation (eVFA ≥100 cm^2^) and that the frequency of two or more as a vascular score is significantly higher in such patients than in non-obese patients without visceral fat accumulation (BMI <25 kg/m^2^ and eVFA <100 cm^2^) (87.5% vs. 42.9%, respectively), while their serum adiponectin levels tended to be lower (5.96 ± 1.9 vs. 10.23 ± 7.14 μg/ml, *P* = 0.09). Thus, the type 2 diabetic patients with visceral fat accumulation, even if they are not obese, might still develop systemic arteriosclerosis and dysregulation of adipocytokines, and are considered to be at high risk of cardiovascular diseases. Asian and Japanese populations could be easily affected with type 2 diabetes mellitus, including those with relatively low BMIs, compared to Caucasians [32], and since many such patients are non-obese, assessment of visceral fat accumulation is important to identify the patients with multiple risk factors of cardiovascular diseases [6,33], and particularly to identify patients who could possibly improve their diabetes and prevent arteriosclerosis through decreasing multiple cardiovascular risk factors by reduction of accumulated visceral fat.

This study found no association between visceral fat accumulation and diabetic microangiopathies, such as retinopathy and nephropathy. Several research groups have reported that glycemic control and the duration of diabetes significantly influence the onset and progression of diabetic microangiopathy [34-36], and herein, the duration of diabetes was significantly shorter in subjects with visceral fat accumulation than in those without, whereas HbA1c levels were not different. Dirani M et al. demonstrated in prospective study that obese diabetic patients were more likely to have diabetic retinopathy [37], while a recent paper reported no significant association between obesity and diabetic retinopathy [38], suggesting that the association between obesity and diabetic microangiopathy seems to be still unclear. Thus, evaluation of microangiopathies should also be carried out, regardless of the patients’ visceral fat accumulation status.

### Eating behaviors and visceral fat accumulation

We also demonstrated different eating behaviors in type 2 diabetic patients with visceral fat accumulation compared to those without visceral fat accumulation, including “food preference”, “eating style”, and “sense of hunger”, and these result were more obvious in male patients. Kozuka et al. [39] reported that hypothalamic endoplasmic reticulum stress was associated with preference for high fat food, which is one of the eating behaviors assessed. Studies from our group have also shown that treatment with liraglutide, a glucagon-like peptide-1 (GLP-1) analogue, improved not only glycemic control, but also obesity, possibly through affecting eating behavior (especially “sense of hunger” and “eating style”) in Japanese type 2 diabetic patients, using the radar chart [20,21]. Glucagon-like peptides have both peripheral effects, such as gastric motility, and central effects, such as inhibition of appetite, through targeting the arcuate nucleus and other hypothalamic lesions [40]. Accordingly, body weight reduction is effective for the treatment of type 2 diabetes with visceral fat accumulation [41], and the reduction of visceral and subcutaneous fat was reported to correlate with the elevation of adiponectin levels [42]. Body weight reduction can be achieved through conventional diet and exercise therapy as well as cognitive therapy [10]. A questionnaire-based radar chart of eating behaviors could therefore be a useful tool for the treatment of type 2 diabetic patients with visceral fat accumulation, by helping patients to visually recognize their eating behaviors and to modify their own eating behaviors by themselves. Although it is possible that there is gender difference in the assessment of eating behaviors using the questionnaire, its etiology remains unclear in the present study.

Taken together, the present results demonstrated the importance of evaluating VFA in type 2 diabetic patients, and for those with visceral fat accumulation, physicians may need to screen for systemic arteriosclerosis more intensively and consider support for patients in modifying their eating behaviors.

This study has several limitations. The study was not prospective in design, included a relatively small population, and was performed in a single institution. The influence of gender, ethnicities, and other residual or unmeasured factors which affect several cardiometabolic factors and eating behaviors cannot be fully excluded. Further prospective studies of larger populations are needed in the future.

## Conclusions

In conclusion, type 2 diabetic patients with visceral fat accumulation showed more advanced systemic arteriosclerosis, lower serum adiponectin levels, and differences in eating behavior, compared to those without visceral fat accumulation.

## Abbreviations

ACS: Acute coronary syndrome
BMI: Body mass index
BP: Blood pressure
CAD: Coronary artery diseases
eGFR: Estimated glomerular filtration rate
ELISA: Enzyme-linked immunosorbent assay
eVFA: Estimated visceral fat area
GLP-1: Glucagon-like peptide-1
HDL-C: High-density lipoprotein cholesterol
IMT: Intima-media thickness
LDL-C: Low-density lipoprotein cholesterol
NDR: No diabetic retinopathy
NGSP: National Glycohemoglobin Standardization Program
PDR: Proliferative diabetic retinopathy
PrePDR: Preproliferative diabetic retinopathy
SDR: Simple diabetic retinopathy
TG: Triglyceride
UA: Uric acid
uACR: Urinary albumin-creatinine ratio
VFA: Visceral fat area
WC: Waist circumference
